# Improvement of Pharmacokinetic Profile of TRAIL via Trimer-Tag Enhances its Antitumor Activity *in vivo*

**DOI:** 10.1038/s41598-017-09518-1

**Published:** 2017-08-21

**Authors:** Haipeng Liu, Danmei Su, Jinlong Zhang, Shuaishuai Ge, Youwei Li, Fei Wang, Michel Gravel, Anne Roulston, Qin Song, Wei Xu, Joshua G. Liang, Gordon Shore, Xiaodong Wang, Peng Liang

**Affiliations:** 1 Department of Biochemistry & Molecular Biology, College of Life Sciences, Sichuan University, Chengdu, China; 2 Laboratory for Therapeutic Development, Rosalind and Morris Goodman Cancer Research Centre, McGill University, Montreal (QC), Canada; 3 Clover Biopharmaceuticals, Chengdu, China; 4 National Institute of Biological Sciences, Beijing, China; 5 GenHunter Corporation, 624 Grassmere Park, Nashville, TN 37211 USA

## Abstract

TNF-related apoptosis-inducing ligand (TRAIL/Apo2L) has long been considered a tantalizing target for cancer therapy because it mediates activation of the extrinsic apoptosis pathway in a tumor-specific manner by binding to and trimerizing its functional receptors DR4 or DR5. Despite initial promise, both recombinant human TRAIL (native TRAIL) and dimeric DR4/DR5 agonist monoclonal antibodies (mAbs) failed in multiple human clinical trials. Here we show that in-frame fusion of human C-propeptide of α1(I) collagen (Trimer-Tag) to the C-terminus of mature human TRAIL leads to a disulfide bond-linked homotrimer which can be expressed at high levels as a secreted protein from CHO cells. The resulting TRAIL-Trimer not only retains similar bioactivity and receptor binding kinetics as native TRAIL *in vitro* which are 4–5 orders of magnitude superior to that of dimeric TRAIL-Fc, but also manifests more favorable pharmacokinetic and antitumor pharmacodynamic profiles *in vivo* than that of native TRAIL. Taken together, this work provides direct evidence for the *in vivo* antitumor efficacy of TRAIL being proportional to systemic drug exposure and suggests that the previous clinical failures may have been due to rapid systemic clearance of native TRAIL and poor apoptosis-inducing potency of dimeric agonist mAbs despite their long serum half-lives.

## Introduction

TNF-related apoptosis-inducing ligand (TRAIL/Apo2L) mediates activation of the extrinsic apoptosis pathway and has long been considered a tantalizing target for cancer therapy based on its ability to selectively kill cancer cells via death receptor (DR) 4 and 5 activation^1–4^. TRAIL is expressed as a homotrimeric type II transmembrane or soluble protein^5,6^. TRAIL-binding induces DR4 and DR5 receptor trimerization, the prerequisite for initiation of apoptotic signaling pathway^7–10^. Upon their trimerization, DR4 and DR5 cytoplasmic domains serve as a docking site for adapter protein Fas-associated death domain (FADD), followed by recruitment of initiator procaspases 8 and 10^7,11^. The resulting assembly of proteins comprise the death-inducing signaling complex (DISC). After caspase 8 autolytic cleavage, activated caspase 8 can then cleave and activate downstream effector caspases 3, 6 and 7^2^.

Various agonists of DR4 and DR5 have been previously produced and described, although development of a viable therapeutic candidate has proven to be challenging. Agonist monoclonal antibodies (mAbs) against DR4 or DR5 have been the most common approach given their long serum half-lives *in vivo*. A number of them have been already tested in human clinical trials^9^, including mapatumumab (HGS-ETR1), lexatumumab (HGS-ETR2), drozitumab (PRO95780), conatumumab (AMG-655), tigatuzumab (CS-1008) and LBY-135. However, none of these agonist mAbs have demonstrated antitumor response rates in patients that warranted advancing their development into pivotal phase III trials^9^. Even in preclinical tumor xenograft models in mice, mapatumumab and lexatumumab failed to shrink tumors, proving to only transiently delay tumor growth^12,13^. The same problem was observed for drozitumab, and it required a crosslinking antibody to enhance its bioactivity^14^. Because DR4 and DR5 receptors must be trimerized upon ligand binding in order to initiate the extrinsic apoptosis pathway, the clinical failures of all agonist mAbs tested to-date is unsurprising, given their inherently dimeric antibody structure.

Utilization of a recombinant human native TRAIL (native TRAIL) would seem to be a more sensible approach because it adopts the natural trimeric structure and full functional potency. Despite initial great promise in preclinical tumor xenograft models in animals^15,16^, Amgen’s native TRAIL (dulanermin) also failed to produce desired antitumor efficacy in human clinical trials, albeit with a good safety profile^9^. The lack of efficacy has been linked to dulanermin’s poor pharmacokinetic profile, as it exhibits a very short half-life in mammals; this is likely due to its small molecular weight and the instability of its non-covalently linked trimeric structure, both ultimately leading to its rapid elimination via renal filtration^15^. Thus, trimerization via covalent bond-linkage may stabilize TRAIL/Apo2L trimeric structure essential for its biological activity as well as increase its molecular weight in order to extend half-life for improved antitumor efficacy *in vivo*.

Although there have been various approaches to overcoming such challenges faced by native TRAIL, such attempts have ultimately fallen short as feasible human therapies. A leucine zipper fused TRAIL^17^ (izTRAIL) and an N-terminus His-tagged TRAIL (SuperKillerTRAIL) stabilized by inserted mutation allowing an additional disulfide bond^18^ are both potentially immunogenic in humans due to either the nonhuman nature of the added leucine-zipper domain or mutations to create inter-chain disulfide bond; moreover, both, and in particular the His-tagged form, have been attributed to hepatotoxicity not observed with native TRAIL^16,19^. Other methods for extending half-life of TRAIL have also encountered obstacles; albumin conjugated TRAIL nanoparticles must be prepared in organic solvents and production was extremely limited^20^. Similar production issues are faced by liposome conjugated TRAIL^21^. PEGylated TRAIL observed accelerated blood clearance phenomenon causing a shortened *in vivo* half-life with repeated dosing^22^. Furthermore, issues related to agent release from carriers are a hindrance for TRAIL encapsulated liposomes and nanoparticles^23,24^.

**Figure 1:**
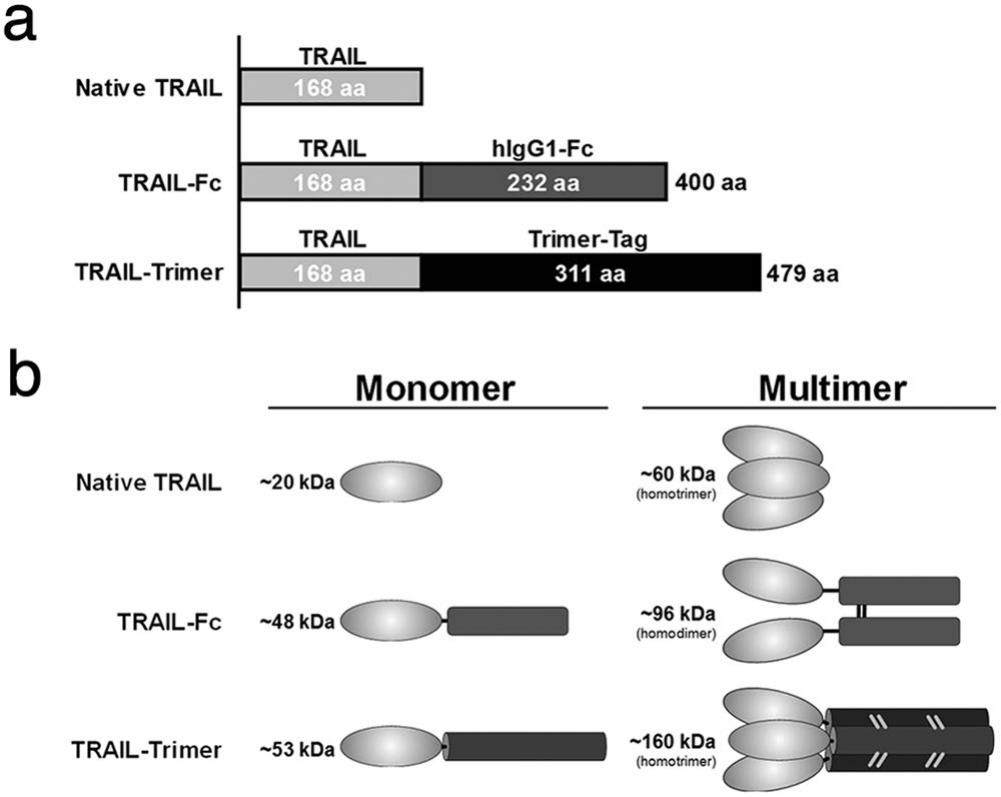
Structures of different TRAIL polypeptides. (**a**) Three recombinant soluble forms of human TRAIL have been used in this study: a native TRAIL comprised of the extracellular domain of TRAIL, a dimeric TRAIL-Fc comprised of extracellular TRAIL domain fused to human IgG1 Fc domain, and a TRAIL-Trimer comprised of extracellular TRAIL domain fused to Trimer-Tag. Amino acid sequence lengths are shown for each protein and domain respectively. (**b**) Theoretical molecular weights (kDa) of both monomeric and multimeric forms of native TRAIL, TRAIL-Fc and TRAIL-Trimer, respectively. Native TRAIL associates into a non-covalently-linked homotrimer, TRAIL-Fc forms a covalently-linked homodimer, and TRAIL-Trimer forms a covalently-linked homotrimer.

In this study, we show that in-frame fusion of human C-propeptide of α1(I) collagen (dubbed as Trimer-Tag) to the C-terminus of mature human TRAIL leads to a disulfide bond-linked homotrimer (Fig. 1) which can be expressed at high levels as a secreted protein from CHO cells. The resulting human TRAIL-Trimer fully retained the naturally potent bioactivity and high receptor binding kinetics of native TRAIL *in vitro* which are 4–5 orders of magnitude superior to that of dimeric DR4 and DR5 agonists. Importantly, TRAIL-Trimer manifested significantly more favorable pharmacokinetic and pharmacodynamic profiles *in vivo* compared to native TRAIL, while preserving a benign safety profile.

## Results

### High-level expression and purification of TRAIL-Trimer fusion protein

**Figure 2:**
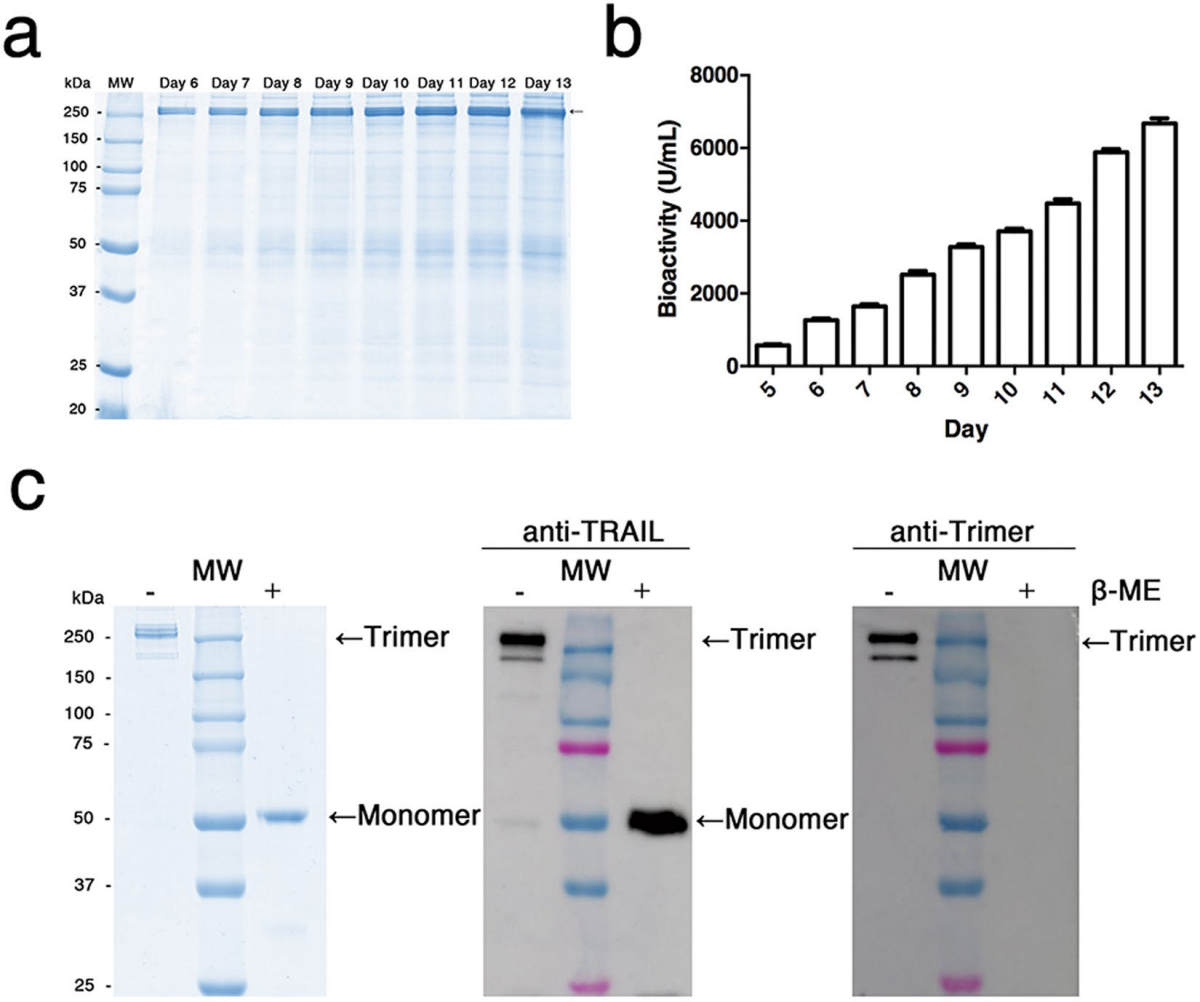
High-level expression and purification of TRAIL-Trimer fusion protein. (**a**) 10% SDS-PAGE analysis of TRAIL-Trimer expression from a fed-batch serum-free cell culture in the bioreactor. 10 µL of cell-free conditioned medium from Day 6 to Day 13 were analyzed under non-reducing condition followed by Coomassie Blue staining. (**b**) Bioassay analysis of TRAIL-Trimer production in conditioned medium from Day 5 to Day 13. (**c**) SDS-PAGE and western blot analysis of purified TRAIL-Trimer under either non-reducing or reducing conditions. 2 µg of purified protein was analyzed by a 10% SDS-PAGE and stained with Coomassie Blue. 0.2 µg of purified protein was analyzed by western blot using monoclonal antibody against TRAIL-domain and Trimer-domain, respectively.

To produce highly pure and sufficient amounts of TRAIL-Trimer fusion protein for functional analyses, we began by screening for high-titer production clones of TRAIL-Trimer vector-transfected CHO cells via MTX-mediated gene amplification; the resulting leading clone was then adapted to serum free media and grown under fed-batch cell culture process in a bioreactor, which led to high-level expression of TRAIL-Trimer (Fig. 2a). During the course of the cell culture process, samples were taken to assess the bioactivity of TRAIL-Trimer using a TRAIL-sensitive human colon cancer derived cell line – COLO205 – by MTT staining (Fig. 2b); as expected, bioactivity increased over time as the production of TRAIL-Trimer continued.

To obtain the TRAIL-Trimer in a highly pure form, TRAIL-Trimer from serum-free conditioned medium was purified to homogeneity by consecutive chromatographic separation steps using Blue Sepharose, Capto Q column and gel filtration. The purified TRAIL-Trimer fusion protein was characterized by SDS-PAGE under either non-reducing or reducing conditions followed by Coomassie blue staining (Fig. 2c, left). The results clearly indicated that TRAIL-Trimer formed a disulfide bond-linked trimer as predicted. Western blot analysis using either a polyclonal antibody detecting human native TRAIL or a monoclonal antibody specific to the Trimer-Tag-domain confirmed the structural feature and integrity of the fusion protein (Fig. 2c, middle and right), which existed essentially as a covalently-linked homotrimer under non-reducing conditions. It was evident that the monoclonal antibody to the Trimer-Tag-domain could only recognize the epitope under the non-reducing condition, consistent with the structure of the antigen used for the immunization to generate the antibody.

### Structural comparison of TRAIL-Trimer, TRAIL-Fc and native TRAIL

**Figure 3:**
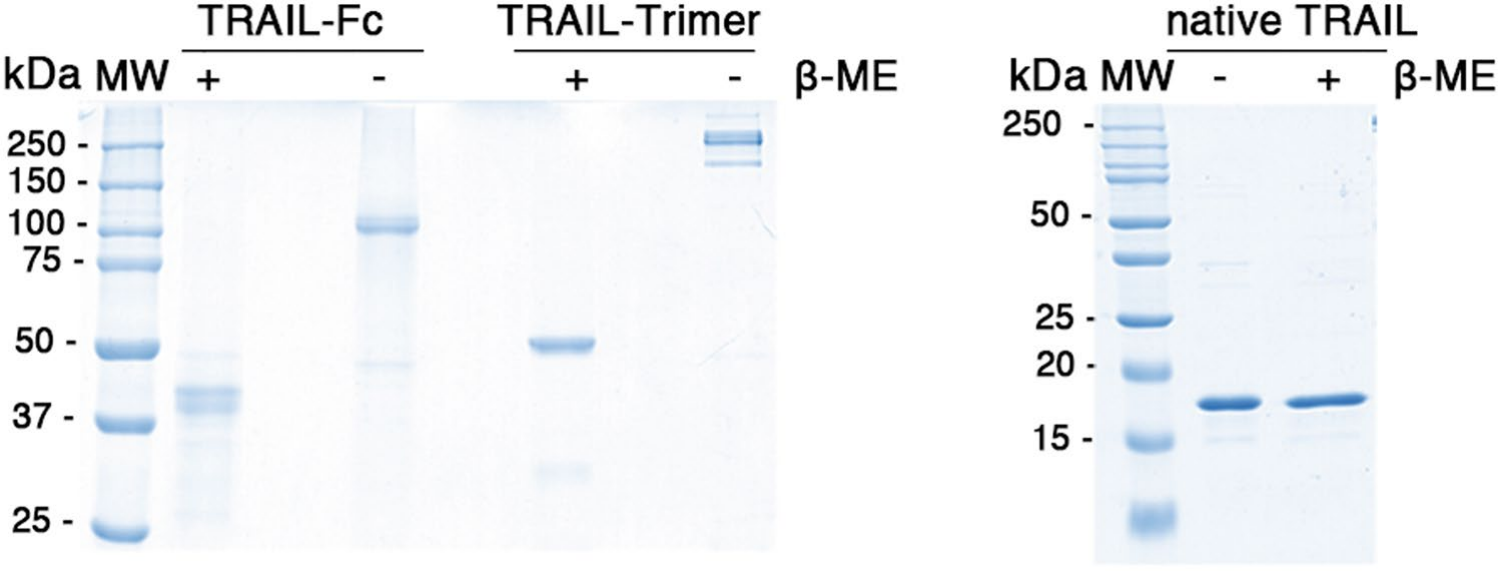
Purity evaluation of TRAIL-Trimer, TRAIL-Fc and native TRAIL. SDS-PAGE analysis of purified TRAIL-Trimer, TRAIL-Fc and native TRAIL under either non-reducing or reducing conditions. 2 µg of purified protein was analyzed by 10% or 15% SDS-PAGE, respectively, and stained with Coomassie Blue.

To systematically compare TRAIL-Trimer with either a dimeric TRAIL and a native TRAIL, we also produced a recombinant TRAIL-Fc fusion protein from CHO cells and native TRAIL from *E. coli* (Fig. 1), following the strategy of dulanermin production^15^. After purification, all three proteins were analyzed first by SDS-PAGE under both non-reducing and reducing conditions to verify their respective structures. The results confirmed that TRAIL-Trimer maintained a homotrimer structure, whereas TRAIL-Fc was a disulfide bond-linked dimer, and native TRAIL was noncovalently-linked (Fig. 3), consistent with previous studies indicating that the homotrimer of native TRAIL is maintained via hydrophobic interfaces on adjacent subunits that are weak and noncovalent in nature^25^.

### Comparison of TRAIL-Trimer, TRAIL-Fc and native TRAIL bioactivity *in vitro*

**Figure 4:**
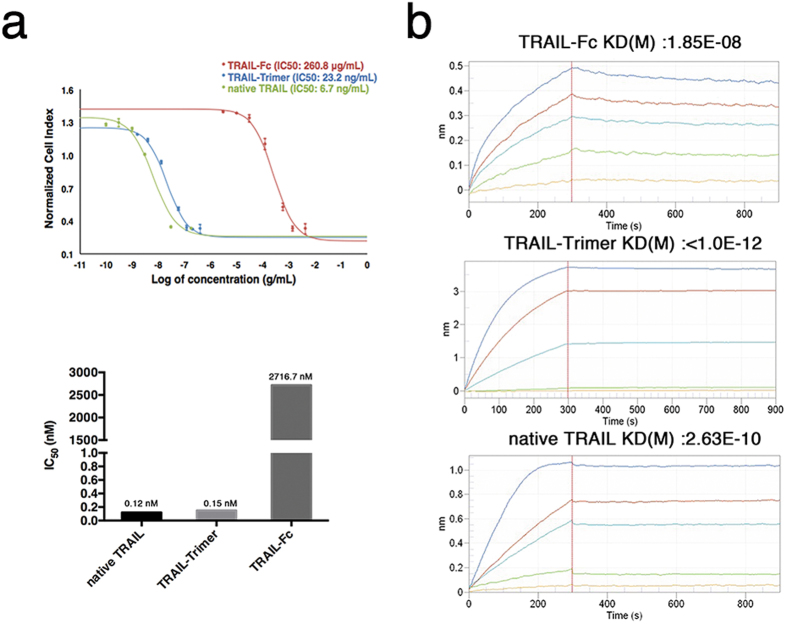
Bioactivity and affinity with DR5-Fc comparison of TRAIL-Trimer, TRAIL-Fc and native TRAIL. (**a**) Bioactivity detection of TRAIL-Trimer, TRAIL-Fc and native TRAIL. The IC_50_ value of TRAIL-Trimer, TRAIL-Fc and native TRAIL were assessed using a TRAIL sensitive cell line COLO205 on a Real-Time Cell Analysis (RTCA) system. The IC_50_ value was obtained according to the dose-response cell index (CI) curve. The IC_50_ value of TRAIL-Trimer, TRAIL-Fc and native TRAIL is 23.2 ng/mL, 260.8 µg/mL, and 6.7 ng/mL, respectively. The bottom graph displays IC_50_ values when molar ratio-adjusted based on theoretical molecular weights for each protein. (**b**) Kinetic parameters of TRAIL-Trimer, TRAIL-Fc and native TRAIL binding to the soluble DR5-Fc fusion protein was assessed by biolayer interferometry measurements. The Super Streptavidin biosensor tips of the ForteBio Octet RED 96 were coated with biotinylated DR5-Fc. The biosensor tips were dipped in increasing concentrations gradient of the same molarity (15.4 nM–123.5 nM) to measure binding of TRAIL-Trimer, TRAIL-Fc and native TRAIL to DR5-Fc and subsequently moved to wells containing buffer (PBS) to measure dissociation rates. Detailed results are shown in Table 1.

The bioactivity IC_50_ values of TRAIL-Trimer, TRAIL-Fc and native TRAIL were assessed using a TRAIL-sensitive human colon cancer cell line – COLO205 – via Real-Time Cell Analysis (RTCA) system. Dose-response curves were generated based on cell viability, and the IC_50_ values were obtained according to the dose-response cell index (CI) curve. COLO205 cells were exposed to increasing concentrations of TRAIL-Trimer, TRAIL-Fc or native TRAIL for 16 hr, and IC_50_ values for TRAIL-Trimer, TRAIL-Fc and native TRAIL were determined to be 23.2 ng/mL, 260.8 µg/mL and 6.7 ng/mL, respectively (Fig. 4a, top). Because the predicted molecular weights of TRAIL-Trimer (~160 kDa) and TRAIL-Fc (~96 kDa) are both significantly larger than native TRAIL (~60 kDa) due to their fused Trimer-Tag and Fc domains respectively (Fig. 1), we then calculated the molar ratio-adjusted IC_50_ values in order to more accurately compare the bioactivities of TRAIL-domains present in each protein. On a molar ratio-adjusted basis, the IC_50_ values for TRAIL-Trimer, TRAIL-Fc and native TRAIL were 0.15 nM, 2716.7 nM and 0.12 nM, respectively (Fig. 4a, bottom). These results demonstrate that the bioactivity of trimeric forms of TRAIL are over 4 orders of magnitude higher than that of dimeric TRAIL. A previous concern for TRAIL-Trimer was if the natural bioactivity of the trimeric TRAIL conformation could be preserved in the fusion protein; the results presented here clearly demonstrate that the bioactivities of TRAIL-Trimer and native TRAIL are equivalent. Additionally, as expected, TRAIL-Trimer induction of apoptosis of COLO205 cells was confirmed at the molecular level via downstream cleavages of procaspase 3, procaspase 8 and PARP (Supplementary Fig. S1).

### Comparison of TRAIL-Trimer, TRAIL-Fc and native TRAIL in receptor binding avidity *in vitro*

DR5 (TRAIL-R2) is one of TRAIL’s functional receptors which initiates extrinsic apoptosis pathway signaling upon activation^1^; thus, we examined the binding kinetic profile of TRAIL-Trimer, TRAIL-Fc and native TRAIL to a soluble DR5-Fc fusion protein. With Fortebio biolayer interferometry measurement, the biotin-labeled DR5-Fc was first captured on Streptavidin (SA) sensors, and real-time binding curves were measured and plotted by applying the sensor in gradient concentrations (15.4 nM-123.5 nM) of the three analytes (Fig. 4b). TRAIL-Trimer was observed to have picomolar binding affinity to DR5-Fc (K_D_ < 1.0 × 10^−12^ M). Unsurprisingly, the dimeric TRAIL-Fc exhibited a receptor binding affinity over 4 orders of magnitude lower (K_D_ = 1.85 × 10^−8^ M) than that of TRAIL-Trimer. Interestingly, the DR5-Fc binding affinity of TRAIL-Trimer was over two orders of magnitude higher than that of native TRAIL (K_D_ of 2.63 × 10^−10^ M). While native TRAIL bound to DR5-Fc (K_on_ = 1.75 × 10^5^ Ms^−1^) twice as fast as TRAIL-Trimer (K_on_ = 8.1 × 10^4^ Ms^−1^), native TRAIL dissociated (K_off_ = 4.6 × 10^−5^ s^−1^) from DR5-Fc at a rate >460 times faster than TRAIL-Trimer (K_off_ < 1 × 10^−7^ s^−1^) (Table 1). These results suggest that the covalent nature of the homotrimeric linkages in TRAIL-Trimer may stabilize the ligand-receptor binding, while the noncovalent trimerization of native TRAIL assumes a conformation more susceptible to dissociation from the receptor.

**Table 1:** Affinity and rate constants of TRAIL-Trimer, TRAIL-Fc and native TRAIL with DR5-Fc.

Analyte	Receptor	K_on_ (1/Ms)	K_off_ (1/s)	KD (M)
TRAIL-Fc	DR5-Fc	9.25E + 03	1.71E-04	1.85E-08
native TRAIL	DR5-Fc	1.75E + 05	4.6E-05	2.63E-10
TRAIL-Trimer	DR5-Fc	8.10E + 04	<1.0E-07	<1.0E-12

### Pharmacokinetic profile of TRAIL-Trimer vs. native TRAIL in mice

**Figure 5:**
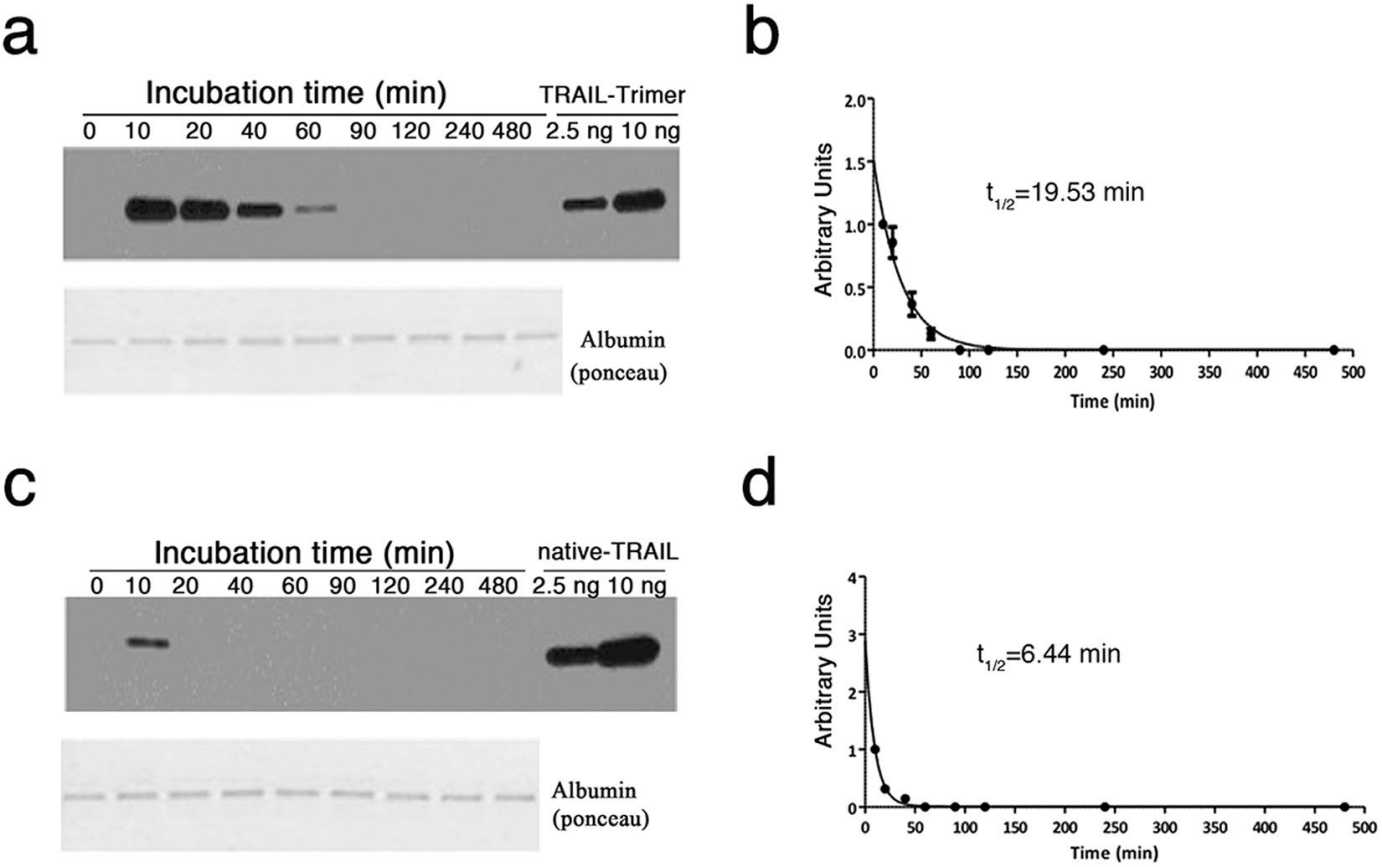
Pharmacokinetic profile of TRAIL-Trimer and native TRAIL determined by western blot. Mice were injected intravenously with equimolar amounts of TRAIL-Trimer (40 mg/kg) or native TRAIL (15 mg/kg) (n = 3 for each protein). (**a**,**c**) Relative serum concentrations of both TRAIL-Trimer (**a**) and native TRAIL (**c**) were evaluated at periodic intervals by western blotting. TRAIL proteins were immunoblotted with an antibody against human TRAIL, and visualized by chemiluminescence. The images were acquired using the ChemiDoc Touch Imaging System (Bio-Rad) and the quantification of the amount of TRAIL proteins present in each sample was done using Image Lab software (Bio-Rad). (**b**,**d**) Estimated half-lives of TRAIL-Trimer (**b**) and native TRAIL (**d**) were calculated using GraphPad Prism v5.04. Error bars represent ± SEM.

We examined the pharmacokinetic profiles for TRAIL-Trimer and native TRAIL; nude mice were injected intravenously (*i.v*.) with equimolar amounts of TRAIL-Trimer (40 mg/kg) or native TRAIL (15 mg/kg), and relative serum concentrations of both proteins were evaluated at periodic intervals by western blot analysis (Fig. 5a,c). TRAIL-Trimer concentration decayed *in vivo* at a much slower rate than that of native TRAIL, with the former able to be visually seen at up to 60 min following *i.v*. injection, while native TRAIL could be detected at low signal up to 10 min. The amounts of TRAIL proteins were quantified using Image Lab software (Bio-rad), and the half-lives of TRAIL-Trimer and native TRAIL were determined to be 19.53 min and 6.44 min respectively (Fig. 5b,d), representing an approximately 3-fold longer half-life for TRAIL-Trimer. This half-life observed for native TRAIL in nude mice is consistent with the ~3–8 min half-life observed in previous studies^15,26^. These results indicate that TRAIL-Trimer is less rapidly eliminated and is more stable than native TRAIL *in vivo*.

### Comparison of antitumor activity of TRAIL-Trimer and native TRAIL *in vivo*

**Figure 6:**
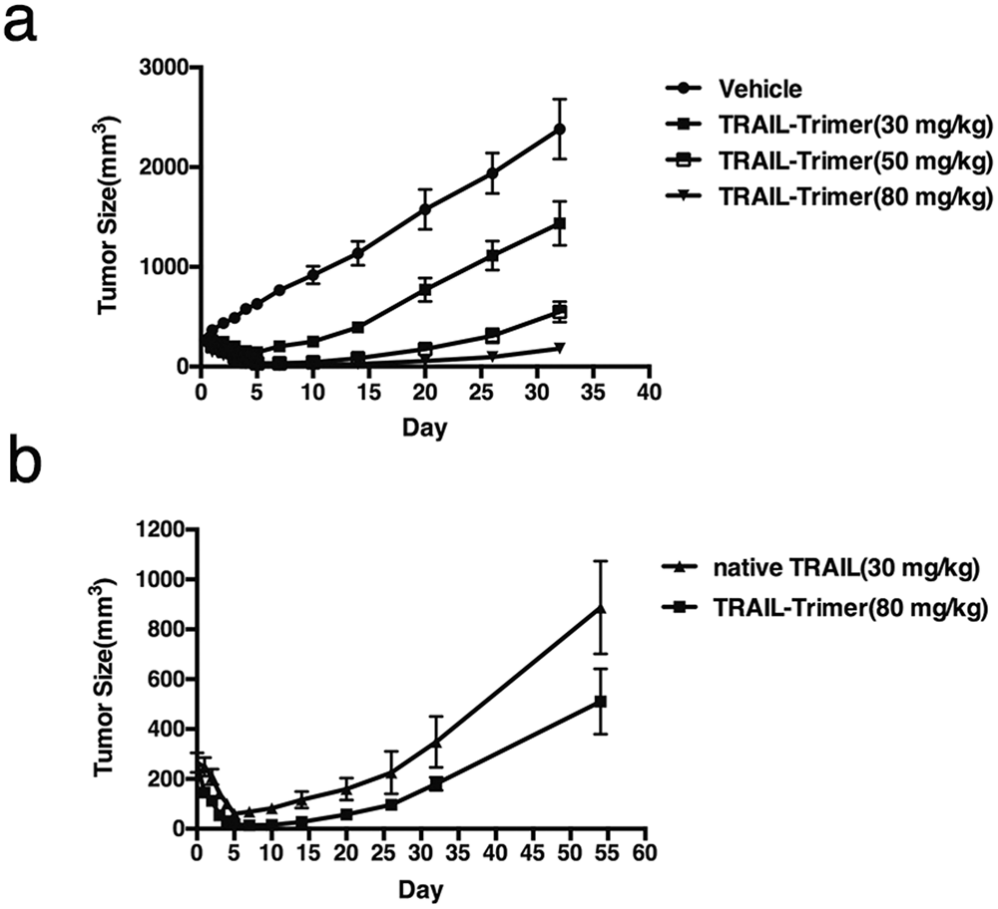
*In vivo* antitumor activities of TRAIL-Trimer and native TRAIL. (**a**) Nude mice with established COLO205 xenografts were given TRAIL-Trimer (30, 50, or 80 mg/kg/day) or vehicle as an i.v. bolus for 5 consecutive days (n = 6/group). Results shown are group mean (±S.D.). (**b**) Nude mice with established COLO205 xenografts were given the same molar concentration of TRAIL-Trimer and native TRAIL. Results shown are group mean (±S.D.).

We then examined the ability of TRAIL-Trimer and native TRAIL to kill human tumor cells *in vivo* by using a common tumor xenograft model in nude mice^12,13,15^. After subcutaneous tumors from inoculated COLO205 human colon cancer cell line had grown to an average size of ~270 mm^3^, mice were randomized (n = 6/group), and TRAIL-Trimer doses of 30 mg/kg, 50 mg/kg or 80 mg/kg were given intravenously once-daily over the first 5 days of the study. Tumor sizes rapidly decreased following TRAIL-Trimer administration in a dose-dependent fashion (Fig. 6a). In contrast, tumors from mice that had been administered with formulation buffer (negative control) grew rapidly and continuously.

The dose-response of TRAIL-Trimer observed here suggests that the best antitumor activity with this regimen is achieved with 80 mg/kg/day, where the longest sustained antitumor response was observed. Thus, we then compared the efficacy of TRAIL-Trimer (80 mg/kg/day) to an equimolar dose of native TRAIL (30 mg/kg/day), given the approximately 2.7 fold difference in molecular weight between the two proteins. At every time point that tumor sizes were measured following dosing, tumors in mice treated with TRAIL-Trimer were smaller than in mice treated with native TRAIL (Fig. 6b), demonstrating that TRAIL-Trimer antitumor activity *in vivo* is superior to native TRAIL in this model. These results are consistent with the superior systemic exposure (pharmacokinetic profile) and receptor-binding affinity results also observed.

The liver cytotoxicity in nude mice injected with TRAIL-Trimer and native TRAIL were also evaluated using histological detection (Supplementary Fig. S2), and neither TRAIL-Trimer nor native TRAIL showed any noticeable toxicities toward the murine liver cells; this apparent lack of hepatotoxicity was further confirmed in normal human liver cells exposed to high levels of TRAIL-Trimer *in vitro* (Supplementary Fig. S3). Nor were there any significant variations in body weights observed across nude mice treatment groups (Supplementary Fig. S4). Additionally, in a separate study in cynomolgus monkeys evaluating various safety serum parameters following TRAIL-Trimer administration, no dose limiting toxic side effects including those on liver and kidney were observed, further confirming the safety profile of TRAIL-Trimer (Supplementary Fig. S5 and Supplementary Table 1).

## Discussion

Validation of TRAIL as an important target in the war against cancer remains to be fully tested, given the suboptimal therapeutic characteristics of previous agonist mAb and native TRAIL approaches. Dimeric agonist mAbs exhibit low activity in inducing apoptosis, and while native TRAIL is highly potent, it is trimerized via weak noncovalent interactions and rapidly eliminated *in vivo*^15,26^.

Utilizing Trimer-Tag to produce a TRAIL-Trimer fusion protein represents a novel and attractive approach to overcoming such obstacles encountered by agonist mAbs and native TRAIL. The shed trimeric C-propeptide of type I collagen from which Trimer-Tag is derived is found in the sera of normal human adults at a concentration in the range of 50–300 ng/mL^27^, and in people with familial high serum concentration of C-propeptide of type I collagen, levels can reach as high as 1–6 μg/mL with no apparent abnormalities, suggesting C-propeptide is not toxic^28^. Structural studies suggest that C-propeptide is indeed a tri-lobed structure with all three subunits coming together in a junction region near their N-termini to connect to the rest of the procollagen molecule^29^, and each trimer is covalently strengthened via 3 pairs of disulfide bonds formed between the neighboring subunit^30^. Although other protein trimerization domains, such as GCN4 from yeast^31^, fibritin from bacteria phage T4^32^ and aspartate transcarbamoylase of *Escherichia coli*^33^, have been described previously to allow trimerization of heterologous proteins, none of these trimerizing proteins are human in nature, nor are they naturally secreted proteins; thus, the fatal drawback of using such non-human protein trimerization domains would be their presumed immunogenicity in humans, rendering such fusion proteins ineffective shortly after administration. Thus, another advantage of Trimer-Tag is that it is derived from a fully human sequence. Drawing a parallel to the evolution in mAb technology, fully human and humanized mAbs have become the gold standard because they typically elicit less immunogenicity than prior chimeric or non-human approaches^34^. Additionally, multiple fusion proteins utilizing fully human IgG Fc domain fused to fully human receptors have been successfully developed in the clinic and are now commercially available therapies^35–37^.

In this first head-to-head study comparing native TRAIL with a dimeric TRAIL-Fc and TRAIL-Trimer fusion proteins, our analysis in bioactivity and receptor binding kinetics corroborate with the observation that DR4/5 must be trimerized in order to fully activate extrinsic apoptosis signaling; indeed, previous studies had indicated that that three DR4/5 receptor subunits are needed to recruit only one FADD molecule^8^. Such results may provide an experimental basis for the clinical failures of all previous dimeric agonist mAbs, as they cannot trimerize and fully activate DR4/5 on their own; in fact, it has been shown that further crosslinking of these mAbs with anti-Fc are required for efficient antibody-induced DISC formation and cell apoptosis^14,38^.

Having established that TRAIL-Fc, and thus predictably any agonist mAbs, are mediocre in death receptor-activating potency despite their long half-lives, we then focused on our head-to-head comparison between TRAIL-Trimer and native TRAIL. We observed serum elimination half-life of TRAIL-Trimer fusion protein in rodents to be > 3 × longer than what was observed for native TRAIL. Because native TRAIL exists as a noncovalently-associated trimer (~60 kDa), it is postulated that its dissociation in the kidney into monomers (~20 kDa) results in its rapid clearance, and its observed clearance rate was similar to the clearance that would be predicted for 20 kDa proteins^15,39^, which may explain the failure of dulanermin in phase II trials despite initial excitement from data in multiple phase I trials^9^. In contrast, TRAIL-Trimer fusion protein exists as a stable covalently-linked trimer, and the larger size of the molecule (~160 kDa) puts it above the limits for elimination by rapid renal filtration.

Prolonged half-life is a critical attribute because it results in greater systemic drug exposure to the target tumor cells, so the short half-life of native TRAIL makes it unlikely able to sufficiently extend beyond the perivascular space before being eliminated^15^, a barrier that TRAIL-Trimer may be able to overcome. Indeed, here TRAIL-Trimer exhibited marked antitumor activity *in vivo* in a dose-dependent manner, with more delayed tumor relapse times even at lower drug concentrations (molar) compared to what was observed in a previous study using native TRAIL in the same tumor xenograph model^15^. Importantly, in our head-to-head comparison using this model, we confirm the prolonged half-life of TRAIL-Trimer versus native TRAIL indeed translated to superior antitumor efficacy. This causal relationship between improved pharmacokinetics and enhanced pharmacodynamic activity has also been previously demonstrated for other biologic cancer therapies^35^.

Notably, TRAIL-Trimer was demonstrated to be safe in both mice and cynomolgus monkeys in our study, without any hepatocyte toxicity. A previous concern with recombinant human TRAIL therapy was that its usefulness to treat tumors *in vivo* may be limited due to apparent hepatotoxicity, although it has been demonstrated that only polyhistidine-tagged TRAIL is hepatotoxic, while non-his-tagged native TRAIL is not^16^. This is consistent with our finding that neither native TRAIL nor TRAIL-Trimer conferred such toxicity.

Further investigation of TRAIL-sensitive tumor types and identification of important biomarkers that would predict either TRAIL sensitivity or resistance may be critical for optimizing the clinical efficacy of TRAIL-Trimer therapy^40^. Several inhibitors of TRAIL-mediated apoptosis are known, such as TRAIL-R3 (decoy receptor 1 (DcR1)) and TRAIL-R4 (DcR2), which embody the extracellular domains of DR4 and DR5 respectively, but have truncated non-functional cytoplasmic death domains^41–43^; indeed, their overexpression on tumors has been shown to inhibit TRAIL-induced apoptosis^44,45^. It has also been shown that loss of proapoptotic proteins Bax and Bak^46^ as well as overexpression of cFLIP^40^ in cancer cells can lead to TRAIL resistance.

Additionally, TRAIL-induced apoptosis may be blocked by overexpression of IAP proteins (IAP-1, IAP-2, XIAP, NIAP, BRUCE and survivin), overexpression of antiapoptotic Bcl-2 proteins (Bcl-2 and Bcl-X_L_) or deficiencies in DNA mismatch repair^47,48^. A small molecule inhibitor of Bcl-2 achieved high response rates in patients with relapsed and refractory Chronic Lymphocytic Leukemia (CLL) and has been approved by the U.S. FDA (Genentech/AbbVie)^49^, validating apoptosis as a target pathway for cancer therapy. Various inhibitors of IAPs (“SMAC mimetics”), have been developed and tested in human clinical trials but have yet to succeed as monotherapies^50^. Given various studies demonstrating that TRAIL therapy may be synergistic with Bcl-2 inhibitors and SMAC mimetics^51,52^, we believe that there is a clear rationale for further investigation into combination therapies with TRAIL-Trimer to further potentiate apoptosis in cancer cells.

The superior pharmacokinetic and pharmacodynamic profiles of TRAIL-Trimer compared to dimeric TRAIL-Fc and native TRAIL represents a significant step-forward for the potential clinical validation of TRAIL as a viable target. Considering the tantalizing, potent tumor-specific killing ability of the TRAIL-induced extrinsic apoptosis pathway, further development of TRAIL-Trimer fusion protein is warranted as a potential advancement in cancer therapy.

## Methods

### Construction of pTRIMER expression vector and expression and purification of TRAIL-Trimer fusion protein

The cDNA sequence encoding the entire human C-propeptide of α1(I) collagen containing 11 glycine-repeat triple helical region was constructed by PCR amplification using an EST clone as a template, purchased from the American Type Culture Collection (ATCC, Manassas, VA, USA). The amplified cDNA encoding the C-propeptide was cloned into the pAPtag-2 mammalian expression vector (GenHunter Corp., Nashville, TN, USA), replacing the AP coding region. The BMP-1 site after the glycine repeats was subsequently mutated from RADD to RNDD (to prevent proteolytic cleavage) using QuikChange kit (Stratagene, Santa Clara, CA, USA), creating the resulting pTRIMER (T0M version) vector. The cDNA encoding the mature human TRAIL was first PCR amplified using an EST clone purchased from ATCC and then cloned between the *HindII* and *Bgl II* sites of the above pTRIMER vector. A short cDNA fragment encoding the signal peptide from human TNFRII was subsequently inserted at the *Hind III site* to allow in-frame fusion to the TRAIL, so TRAIL-Trimer is expressed as a secreted protein. pTRAIL-Trimer expression vector was stably transfected into GH-CHO (dhfr −/−) cell line (GenHunter Corp.) using FUGENE 6 (Roche, Mannheim, Germany) grown in IMDM medium with 10% FBS and 1% Penn-Strep supplemented with HT (Sigma, St Louis, MO, USA). After stepwise gene amplification with increasing concentrations (0.0–0.5 μM) of MTX (Sigma), the clone producing the highest TRAIL-Trimer titer, determined by bioassay with a TRAIL sensitive cell line COLO205 (ATCC), was obtained. The cells were then adapted to SFM-4-CHO (Hyclone, Logan, UT, USA) serum free medium, and TRAIL-Trimer was produced in a 10 L Celligen bioreactor (Eppendorf, Ontario, Canada) under a fed-batch process with CellBoost 2 supplement (Hyclone) added every other day from day 3 until harvest on day 13. TRAIL-Trimer titer was monitored daily using SDS-PAGE. After completion of the upstream cell culture process, TRAIL-Trimer was purified to homogeneity by consecutive chromatographic separation steps on Blue Sepharose (GE Healthcare, Logan, UT, USA), Capto Q column (GE Healthcare) and Superdex 200 (GE Healthcare) per manufacturers’ instructions. The purity of TRAIL-Trimer was determined by both SDS-PAGE and SEC-HPLC (Sepax Zenix-C SEC. 300,Newark, DE, USA).

### Expression and purification of TRAIL-Fc and DR5-Fc fusion proteins

Human TRAIL-Fc and DR5-Fc expression vectors were generated by PCR amplification of the corresponding cDNA templates followed by cloning them into pGH-Fc expression vector (GenHunter Corp.). These vectors were then stably transfected into GH-CHO (dhfr −/−) cell lines (GenHunter Corp.) and high expression clones were selected and adapted to SFM-4-CHO (Hyclone) serum free medium as described for TRAIL-Trimer. TRAIL-Fc and DR5-Fc were purified to homogeneity from the conditioned medium using MabSelect Sure (GE Healthcare) and Superdex 200 (GE Healthcare) according to manufacturer’s instructions. Purified TRAIL-Fc and DR5-Fc were ultra-filtrated into PBS before being used for further characterization.

### Expression and purification of native TRAIL

Native TRAIL expression vector, pQE-nTR, was purchased from Addgene (Cambridge, MA, USA) and contains the cDNA sequence corresponding to the extracellular portion of the human TRAIL (aa 114–281). Recombinant native TRAIL was expressed in transformed DH10β bacteria as the expression host, and purified following the procedure previously described^53^. Protein concentration was determined using Bio-Rad (Hercules, CA, USA) protein assay solution and the purity of the recombinant TRAIL protein was confirmed by SDS-PAGE and Coomassie Blue gel staining. To remove endotoxins, the TRAIL protein preparation was incubated 2 h at room temperature with the endotoxin removal resin (cat. 88276, Thermo Fisher Scientific, Waltham, MA, USA) following manufacturer’s instructions. After elution, the endotoxin concentration was determined using the LAL chromogenic endotoxin quantitation kit (Thermo Fisher Scientific, cat. 88282). Native TRAIL was aliquoted and stored at −80 °C prior to use for further characterization.

### Antibodies and western blots

Antibodies used for this study were: 12B11D11 and 25E11E11 (Clover Biopharmaceuticals, Chengdu, China) which recognize the Trimer-Tag domain and the TRAIL-domain of TRAIL-Trimer fusion protein, respectively. Protein concentrations were determined using a Pierce BCA Protein Assay Kit (Thermo-Fisher Scientific). Purified TRAIL-Trimer (0.2 µg) was analyzed by western blot on a 10% SDS-PAGE under reducing (+β-mercaptoethanol) or nonreducing (-β-mercaptoethanol) conditions using the above two antibodies, followed by goat anti-mouse IgG-HRP (Southern Biotech, Birmingham, AL, USA). Reactive proteins were visualized with an ECL kit following the manufacturer’s protocol. Antibodies against Caspase-8, Caspase-3 and PARP were purchased from Cell Signaling Technology (Danvers, MA, USA), and actin antibody A2066 (Sigma). Secondary antibodies were purchased from Southern Biotech (Birmingham, AL, USA).

### Binding kinetics and affinity measurements

All binding kinetics measurements were performed using Bio-Layer Interferometry on FortéBio Octet QK^e^ instrument (Pall, New York, NY, USA). Prior to kinetics measurements, DR5-Fc was biotinylated with NHS-PEG4-biotin kit (Thermo Fisher Scientific) following the manufacturer’s instructions. Unbound NHS-PEG4-biotin was removed via ultra-filtration in PBS. The protein concentration of biotinylated DR5-Fc was determined by Pierce BCA Protein Assay Kit (Thermo Fisher Scientific). All interaction analyses were conducted at 25 °C in PBS buffer unless stated otherwise. For kinetic analysis of biotinylated DR5-Fc with TRAIL-Trimer, TRAIL-Fc and native TRAIL, soluble biotinylated DR5-Fc (10 µg/mL) were immobilized on streptavidin (SA) biosensors (Pall) for 300 s to ensure saturation. The 96-well microplates used in the Octet were filled with 200 µL of sample or buffer per well. Following a washing step, association and dissociation measurements were carried out using serial dilutions of purified TRAIL-Trimer, TRAIL-Fc and native TRAIL, respectively. Kinetic parameters (K_on_ and K_off_) and affinities (K_D_) were analyzed using Octet software, version 9.0 (Pall).

### RTCA analysis

Bioactivity of purified TRAIL-Trimer, TRAIL-Fc and native TRAIL was evaluated using a Real-Time Cell Analysis (RTCA) iCELLigence system (ACEA Biosciences, San Diego, CA, USA) based on electrical impedance measurement which is a label-free approach for cell-based assays^54^. The impedance data were analyzed and processed by integrated software^55^. The RTCA iCELLigence system was placed in a humidified incubator at 37 °C and 5% CO_2_ conditions. Initially, background of the 8-wells E-Plates was determined in 150 µL RPMI-1640, and subsequently, each well of the E-Plate was added with 300 µL COLO205 cell suspension at cell density of 200,000/well. When the cell index (CI) reached 1 (approximately 24 h of incubation), 50 µL of the purified TRAIL-Trimer, TRAIL-Fc or native TRAIL were added to the corresponding wells to reach final concentrations of 1.6 ng/mL–400 ng/mL, 3 µg/mL–4.2 mg/mL and 0.1 ng/mL–180 ng/mL, respectively. Impedance was measured for at least another 20 h every 15 min, and the normalized cell index (NCI) versus time were plotted on graphs displaying kinetic curves of COLO205 survival rate over time when treated with each fusion protein. IC_50_ values were calculated from the sigmoidal dose response curves by RTCA software^56^.

### TRAIL Bioassay

TRAIL-Trimer bioactivity was assessed by MTT staining. Briefly, COLO205 cells were plated at a density of 40,000 cells/well into 96-well tissue culture plates (Corning Costar, New York, NY, USA). After overnight growth, COLO205 cells were treated with two-fold serial dilutions of TRAIL-Trimer-containing samples for 16 h. Then, 20 µL of the tetrazolium compound MTT (3-(4,5-dimethylthiazol-2-yl)-2,5-diphenyltetrazolium bromide) were added to each well. After incubation for 4 h at 37 °C, the supernatant was removed and formazan crystals were dissolved by DMSO (100 µL/well). Finally, absorbance (490 nm) was measured by Multiskan GO Microplate Spectrophotometer (Thermo Fisher Scientific).

### Pharmacokinetics study and analysis in nude mice

Athymic nude female mice were obtained from Charles River Laboratories (Wilmington, MA, USA). Mouse body weights ranged from 21.4 to 27.7 g. Each mouse was administered with TRAIL-Trimer or native TRAIL via tail vein at equimolar doses of 40 mg/kg and 15 mg/kg, respectively (n = 3/group). Blood samples (25–30 µL) were collected at 10, 20, 40, 60, 90, 120, 240 and 480 min from each animal using heparinized tips. The samples were transferred to a 0.5 mL microcentrifuge tube and plasma separated from each blood sample by centrifugation. All the plasma samples were stored at −80 °C. Blood samples corresponding to T0 were collected the day before injection and processed as described above.

Recovery of TRAIL-Trimer and native TRAIL was measured by western blot. To obtain good sensitivity of detection, plasma samples from mice treated with TRAIL-Trimer or native TRAIL were diluted 1:1100 in 3× SDS-PAGE sample buffer. After heating at 95 °C for 5 minutes, the diluted samples (10 µL) were resolved by SDS-PAGE and analyzed by western blotting. Proteins were separated on a 4–20% SDS-polyacrylamide gel and transferred to nitrocellulose membrane. Membranes were first blocked in 5% skim-milk in TBST and incubated overnight at 4 °C with the goat anti-human TRAIL (1:2000 dilution). After several washes in TBST, membranes were incubated for 1 hour at room temperature with HRP conjugated Trueblot anti-goat IgG (1:2000 dilution) (cat. 18-8814-33, Rockland, Pottstown, PA, USA). Membranes were washed thrice and developed by chemiluminescence. The images were acquired using the ChemiDoc Touch Imaging System (Bio-Rad) and the quantification of the amount of TRAIL proteins present in each sample was done using Image Lab software (Bio-Rad).

### COLO205 tumor xenograft experiments in nude mice

Female nude mice were purchased from Beijing HFK Bioscience Co, Ltd. (Beijing, China) and kept under standard pathogen-free conditions in the animal care center at Sichuan University and received human care. All animal experiments were approved by The Institutional Animal Care and Use Committee (IACUC) in Sichuan University and were conducted according to international guidelines for animal experimentation. For pharmacodynamic (PD) studies, 5 × 10^6^ cells from log phase COLO205 human colon cancer cells were inoculated subcutaneously in the right dorsal flank of each mouse. Tumor volume was calculated by the following equation: tumor volume (mm^3^) = length × width_1_ × width_2_ × 0.5^57^. Six days following inoculation, animals (n = 30) were randomized by tumor size into five groups of 6 mice each (average tumor volume ~270 mm^3^/group). Mice were then given once-daily i.v. bolous doses for 5 consecutive days of TRAIL-Trimer (30, 50, 80 mg/kg/Day), native TRAIL (30 mg/kg/Day), or control vehicle via a tail vein. Tumor volumes were monitored up to 54 days after the first drug-injection in the treatment groups. The body weight of mice was also monitored.

The liver tissues for toxicity evaluation were obtained from mice in the 5 groups, 24 h after the fifth administration of drug. After being fixed with 10% neutral-buffered formalin and paraffin-embedded, sections (5 µm) were cut from liver tissues. The liver sections were stained with hematoxylin and eosin (H&E) for the morphological examination of tissue cells. All tissue-staining images were captured with an upright microscope (BX53, Olympus, Japan).

### Human hepatotoxicity assay

The normal human liver cell line, LO2, was purchased from Shanghai Institute of Cell Biology (Shanghai, China) and cultured in RPMI-1640 with 10% FBS and 2 mmol/L glutamine. LO2 cells were incubated with various concentrations of TRAIL-Trimer at 37 °C for 24 hours, and cell viability was determined using tetrazolium (MTT) colorimetric test. Representative images were taken by phase contrast microscopy, and presented data were from representative experiments of at least 3 independent assays. Cleavage of PARP in LO2 cells was measured by western blot.

## Electronic supplementary material

Supplementary Information

## References

[1] Ashkenazi A, Dixit VM (1998). Death receptors: signaling and modulation. Science.

[2] Wang S, El-Deiry W (2003). S. TRAIL and apoptosis induction by TNF-family death receptors. Oncogene.

[3] Younes M, Georgakis GV, Rahmani M, Beer D, Younes A (2006). Functional expression of TRAIL receptors TRAIL-R1 and TRAIL-R2 in esophageal adenocarcinoma. Eur J Cancer.

[4] Koornstra JJ (2003). Expression of TRAIL (TNF-related apoptosis-inducing ligand) and its receptors in normal colonic mucosa, adenomas and carcinomas. J Pathol.

[5] Wiley SR (1995). Identification and characterization of a new member of the TNF family that induces apoptosis. Immunity.

[6] Pitti RM (1996). Induction of apoptosis by Apo-2 Ligand, a new member of the tumor necrosis factor receptor family. J Biol Chem.

[7] Walczak H, Krammer PH (2000). The CD95 (APO-1/Fas) and the TRAIL (APO-2L) apoptosis systems. Ex Cell Res.

[8] Dickens LS (2012). A death effector domain chain DISC model reveals a crucial role for caspase-8 chain assembly in mediating apoptotic cell death. Mol Cell.

[9] Lemke J, von Karstedt S, Zinngrebe J, Walczak H (2014). Getting TRAIL back on track for cancer therapy. Cell Death Differ.

[10] Ravi R (2001). Regulation of death receptor expression and TRAIL/Apo2L-induced apoptosis by NF-κB. Nat Cell Biol.

[11] Fulda S, Debatin KM (2006). Extrinsic versus intµrinsic apoptosis pathways in anticancer chemotherapy. Oncogene.

[12] Pukac L (2005). HGS-ETR1, a fully human TRAIL-receptor 1 monoclonal antibody, induces cell death in multiple tumour types *in vitro* and *in vivo*. Br J Cancer.

[13] Marini P (2006). Combined treatment of colorectal tumours with agonistic TRAIL receptor antibodies HGS-ETR1 and HGS-ETR2 and radiotherapy: enhanced effects *in vitro* and dose-dependent growth delay *in vivo*. Oncogene.

[14] Wilson NS (2011). An Fcgamma receptor-dependent mechanism drives antibody-mediated target-receptor signaling in cancer cells. Cancer Cell.

[15] Kelley SK (2001). Preclinical studies to predict the disposition of Apo2L/Tumor necrosis factor-related apoptosis-inducing ligand in humans: characterization of *in vivo* efficacy, pharmacokinetics, and safety. JPET.

[16] Lawrence D (2001). Differential hepatocyte toxicity of recombinant Apo2L/TRAIL versions. Nat Med.

[17] Walczak K (1999). Tumoricidal activity of tumor necrosis factor-related apoptosis-inducing ligand *in vivo*. Nat Med.

[18] Wang Y (2004). Synthetic lethal targeting of MYC by activation of the DR5 death receptor pathway. Cancer Cell.

[19] Ashley DM (2008). *In vitro* sensitivity testing of minimally passaged and uncultured gliomas with TRAIL and/or chemotherapy drugs. Br J Cancer.

[20] Bae S (2012). Doxorubicin-loaded human serum albumin nanoparticles surface-modified with TNF-related apoptosis-inducing ligand and transferrin for targeting multiple tumor types. Biomaterials.

[21] Mitchell MJ, Wayne E, Rana K, Schaffer CB, King MR (2014). TRAIL-coated leukocytes that kill cancer cells in the circulation. Proc Natl Acad Sci USA.

[22] Xu H, Wang KQ, Deng YH, Chen DW (2010). Effects of cleavable PEG-cholesterol derivatives on the accelerated blood clearance of PEGylated liposomes. Biomaterials.

[23] Kim TH (2011). PEGylated TNF-related apoptosis-inducing ligand (TRAIL)-loaded sustained release PLGA microspheres for enhanced stability and antitumor activity. J Control Release.

[24] Lim SM (2011). Improved biological half-life and anti-tumor activity of TNF-related apoptosis-inducing ligand (TRAIL) using PEGexposed nanoparticles. Biomaterials.

[25] Won EY (2010). The structure of the trimer of human 4-1BB ligand is unique among members of the tumor necrosis factor superfamily. J Biol Chem.

[26] Xiang H, Nguyen CB, Kelley SK, Dybdal N, Escandón E (2004). Tissue distribution, stability, and pharmacokinetics of Apo2 ligand/tumor necrosis factor-related apoptosis-inducing ligand in human colon carcinoma COLO205 tumor-bearing nude mice. Drug Metab Dispos.

[27] Melkko J, Niemi S, Risteli L, Risteli J (1990). Radioimmunoassay of the carboxyterminal propeptide of human type I procollagen. Clin Chem.

[28] Sorva A (1994). Familial high serum concentrations of the carboxyl-terminal propeptide of type I procollagen. Clin Chem.

[29] Bernocco S (2001). Biophysical characterization of the C-propeptide trimer from human procollagen III reveals a tri-lobed structure. J Biol Chem.

[30] Bourhis JM (2012). Structural Basis of Fibrillar Collagen Trimerization and Related Genetic Disorders. Nat Struct Mol Biol.

[31] Yang X, Farzan M, Wyatt R, Sodroski J (2000). Characterization of stable, soluble trimers containing complete ectodomains of human immunodeficiency virus type 1 envelope glycoproteins. J Virol.

[32] Frank S (2001). Stabilization of short collagen-like triple helices by protein engineering. J Mol Biol.

[33] Chen B (2004). A chimeric protein of simian immunodeficiency virus envelope glycoprotein gp140 and Escherichia coli aspartate transcarbamoylase. J Virol.

[34] Harding FA, Stickler MM, Razo J, DuBridge RB (2010). The immunogenicity of humanized and fully human antibodies. MAbs.

[35] Holash J (2002). VEGF-trap: a VEGF blocker with potent antitumor effects. Proc Natl Acad Sci USA.

[36] Genovese MC (2005). Abatacept for rheumatoid arthritis refractory to tumor necrosis factor α inhibition. N Engl J Med.

[37] Kareskog L (2004). Therapeutic effect of the combination of etanercept and methotrexate compared with each treatment alone in patients with rheumatoid arthritis: double-blind randomised controlled trial. The Lancet.

[38] Haynes NM (2010). CD11c+ dendritic cells and B cells contribute to the tumoricidal activity of anti-DR5 antibody therapy in established tumors. J Immunol.

[39] Clark R (1996). Long-acting growth hormones produced by conjugation with polyethylene glycol. J Biol Chem.

[40] Zhang L, Fang B (2005). Mechanisms of resistance to TRAIL-induced apoptosis in cancer. Cancer Gene Ther.

[41] Pan G (1997). An antagonist decoy receptor and a death domain-containing receptor for TRAIL. Science.

[42] Sheridan JP (1997). Control of TRAIL-induced apoptosis by a family of signaling and decoy receptors. Science.

[43] Ashkenazi A, Dixit VM (1999). Apoptosis control by death and decoy receptors. Curr Opin Cell Biol.

[44] Merino D (2006). Differential inhibition of TRAIL-mediated DR5-DISC formation by decoy receptors 1 and 2. Mol Cell Biol.

[45] Morizot A (2011). Chemotherapy overcomes TRAIL-R4-mediated TRAIL resistance at the DISC level. Cell Death Differ.

[46] LeBlanc H (2002). Tumor-cell resistance to death receptor-induced apoptosis through mutational inactivation of the proapoptotic Bcl-2 homolog Bax. Nat Med.

[47] Roy N, Deveraux QL, Takahashi R, Salvesen GS, Reed JC (1997). The c-IAP-1 and c-IAP-2 proteins are direct inhibitors of specific caspases. EMBO J.

[48] Deveraux QL, Reed JC (1999). IAP family proteins–suppressors of apoptosis. Genes Dev.

[49] Seymour JF (2013). Bcl-2 inhibitor ABT-199 (GDC-0199) monotherapy shows anti-tumor activity including complete remissions in high-risk relapsed/refractory (R/R) chronic lymphocytic leukemia (CLL) and small lymphocytic lymphoma (SLL). Blood.

[50] Bai L, Smith DC, Wang S (2014). Small-molecule SMAC mimetics as new cancer therapeutics. Pharmacol Ther.

[51] Li L (2004). A small molecule Smac mimic potentiates TRAIL- and TNFalpha-mediated cell death. Science.

[52] Fulda S, Meyer E, Debatin KM (2002). Inhibition of TRAIL-induced apoptosis by Bcl-2 overexpression. Oncogene.

[53] Kim KJ (2004). Two-promoter vector is highly efficient for overproduction of protein complexes. Protein Sci.

[54] Ke N, Wang X, Xu X, Abassi YA (2011). The xCELLigence system for real-time and label-free monitoring of cell viability. Methods Mol Biol.

[55] Atienza JM, Zhu J, Wang X, Xu X, Abassi Y (2005). Dynamic monitoring of cell adhesion and spreading on microelectronic sensor arrays. J Biomol Screen.

[56] Ceriotti L (2007). Real-time assessment of cytotoxicity by impedance measurement on a 96-well plate. Sens Actuators B.

[57] Corbert, T. *et al*. *In vivo* methods for screening and preclinical testing: use of rodent solid tumors for drug discovery in *Anticancer Drug Development Guide: Preclinical* Screening, Clinical *Trials and Approval* (ed. Teicher, B.) 75–99 (Humana Press, 1997).

