# Author Correction: Improvement of Pharmacokinetic Profile of TRAIL via Trimer-Tag Enhances its Antitumor Activity *in vivo*

**DOI:** 10.1038/s41598-018-23057-3

**Published:** 2018-03-22

**Authors:** Haipeng Liu, Danmei Su, Jinlong Zhang, Shuaishuai Ge, Youwei Li, Fei Wang, Michel Gravel, Anne Roulston, Qin Song, Wei Xu, Joshua G. Liang, Gordon Shore, Xiaodong Wang, Peng Liang

**Affiliations:** 1 Department of Biochemistry & Molecular Biology, College of Life Sciences, Sichuan University, Chengdu, China; 2 Laboratory for Therapeutic Development, Rosalind and Morris Goodman Cancer Research Centre, McGill University, Montreal (QC), Canada; 3 Clover Biopharmaceuticals, Chengdu, China; 4 National Institute of Biological Sciences, Beijing, China; 5 GenHunter Corporation, 624 Grassmere Park, Nashville, TN 37211 USA

Correction to: *Scientific Reports* 10.1038/s41598-017-09518-1, published online 21 August 2017

In the original version of this Article, there were errors in Affiliation 1 which was incorrectly listed as ‘Department of Biochemistry & Molecular Biology, School of Life Sciences, Sichuan University, Chengdu, China.’ The correct affiliation is listed below:

Department of Biochemistry & Molecular Biology, College of Life Sciences, Sichuan University, Chengdu, China.

These errors have now been corrected in the PDF and HTML versions of the Article.

