# Supplementary material for: Improvement of Pharmacokinetic Profile of TRAIL via Trimer-Tag Enhances its Antitumor Activity in vivo
Source: Sci Rep. 2017 Aug 21;7:8953. doi: 10.1038/s41598-017-09518-1 (PMC5566391; doi:10.1038/s41598-017-09518-1)
Supplement: Supplementary file 1 — Supplementary Information [file 41598_2017_9518_MOESM1_ESM.pdf]

Supplementary Information

Improvement of Pharmacokinetic Profile of TRAIL via Trimer-Tag Enhances its Antitumor Activity *in vivo*

Haipeng Liu, Danmei Su, Jinlong Zhang, Shuaishuai Ge, Youwei Li, Fei Wang, Michel Gravel, Anne Roulston, Qing Song, Wei Xu, Joshua G. Liang, Gordon Shore, Xiaodong Wang, Peng Liang

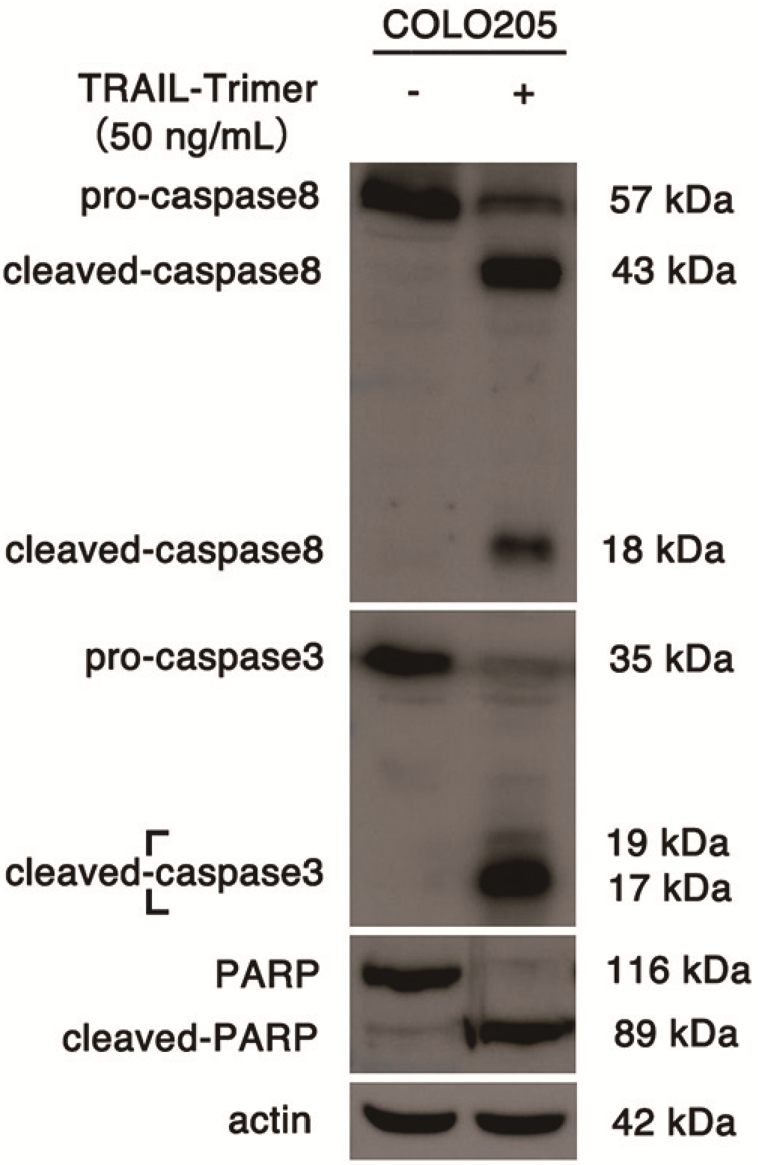

Supplementary Fig. 1 Western blot analysis of procaspase 8, procaspase 3 and PARP cleavage levels after COLO205 cells were exposed to TRAIL-Trimer in vitro.

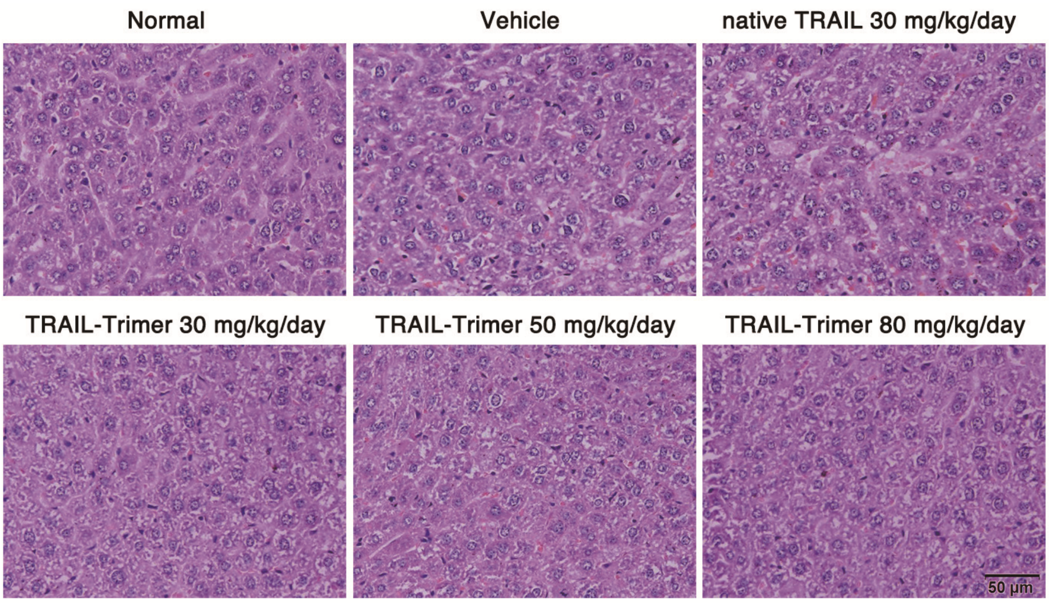

Supplementary Fig. 2 Hepatotoxicity assessment for TRAIL-Trimer treatment in nude mice using histological detection.

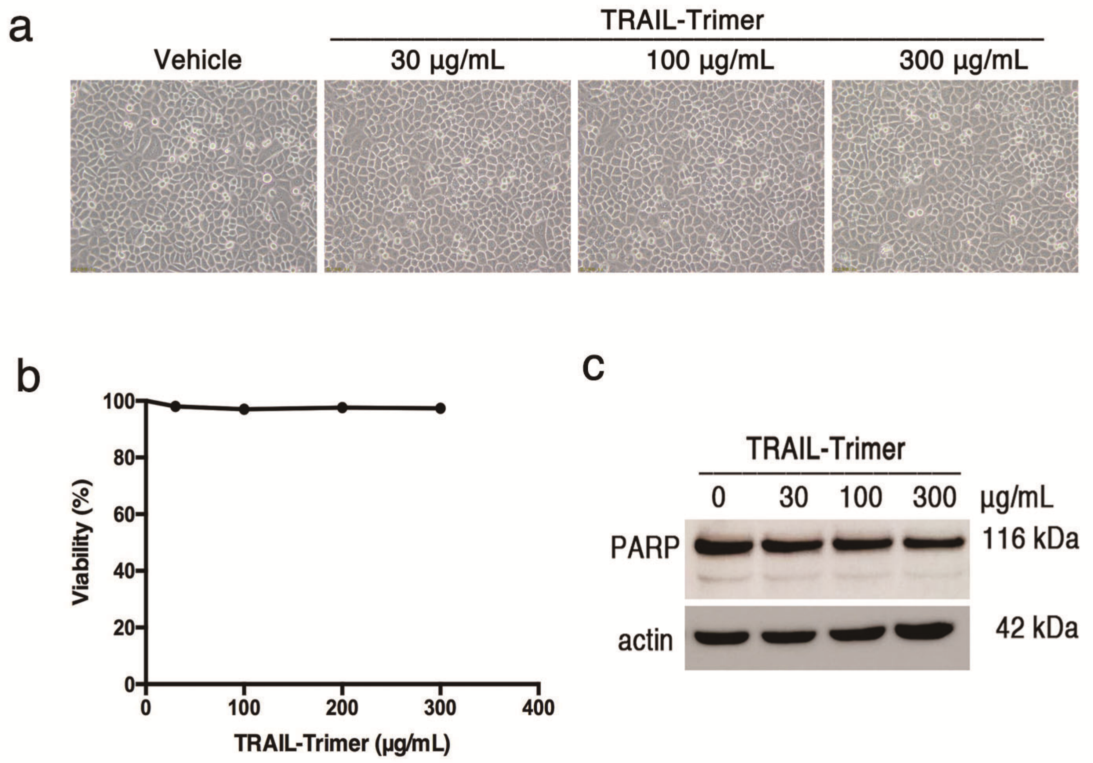

Supplementary Fig. 3 Evaluation of TRAIL-Trimer hepatotoxicity in human normal liver cells (LO2).

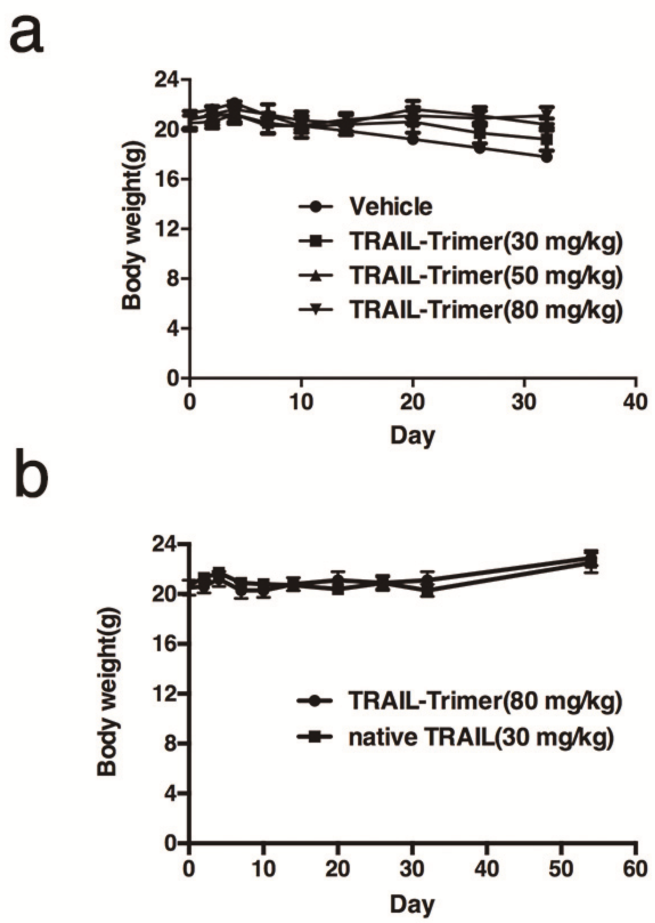

Supplementary Fig. 4 Body weight of each mouse was measured periodically. Results are expressed as mean ± S.D.

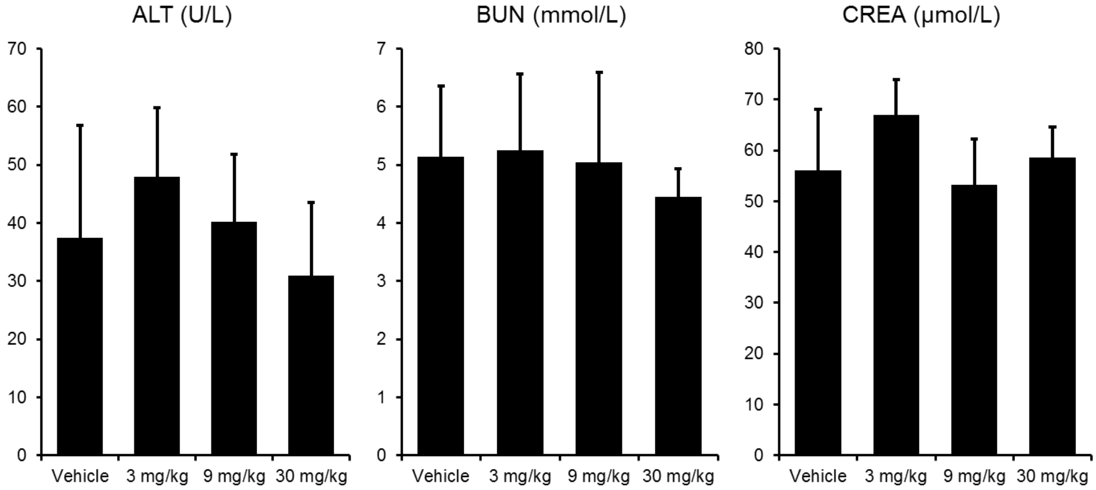

Supplementary Fig. 5 Alanine aminotransferase (ALT), blood urea nitrogen (BUN) and creatinine (CREA) analyses in monkeys after TRAIL-Trimer administration. Results are expressed as mean ± SD (n= 5/group).

Supplementary Table 1: Routine Blood Tests in Monkeys After TRAIL-Trimer Administration (expressed as mean ± SD, n=5/group)

Items	Vehicle	3 mg/kg	9 mg/kg	30 mg/kg
RBC ($x10^6/\mu L$)	4.55 ± 0.26	4.37 ± 0.17	4.17 ± 0.40	4.42 ± 0.32
WBC ($x10^3/\mu L$)	10.44 ± 3.00	11.11 ± 3.57	14.06 ± 5.26	10.51 ± 5.12
PLT ($x10^3/\mu L$)	405.6 ± 104.94	454.60 ± 85.70	364.50 ± 54.49	495.20 ± 63.01
HGB (g/dL)	10.98 ± 0.54	10.82 ± 0.91	10.00 ± 0.76	10.16 ± 0.48
HCT (%)	36.92 ± 2.07	35.28 ± 2.87	32.83 ± 2.07	34.68 ± 2.06
MCH (pg)	24.14 ± 1.28	24.72 ± 1.33	24.00 ± 0.74	23.06 ± 1.14
MCHC (g/dL)	29.70 ± 0.85	30.60 ± 0.20	30.45 ± 0.68	29.38 ± 0.55
MCV (fL)	81.30 ± 5.05	80.70 ± 4.18	78.85 ± 2.91	78.50 ± 3.29
